# Vulnerability assessments, identity and spatial scale challenges in disaster-risk reduction

**DOI:** 10.4102/jamba.v7i1.201

**Published:** 2015-11-30

**Authors:** Edward R. Carr, Daniel Abrahams, Arielle T. de la Poterie, Pablo Suarez, Bettina Koelle

**Affiliations:** 1 Department of International Development, Community, and Environment, Clark University, United States; 2 Society, Environment, Economy Group, LLC, Columbia, SC United States; 3 Department of Geography, University of South Carolina, United States; 4 Environmental Studies Program, University of Colorado, United States; 5 Red Cross Red Crescent Climate Centre, the Hague, the Netherlands

## Abstract

Current approaches to vulnerability assessment for disaster-risk reduction (DRR) commonly apply generalised, a priori determinants of vulnerability to particular hazards in particular places. Although they may allow for policy-level legibility at high levels of spatial scale, these approaches suffer from attribution problems that become more acute as the level of analysis is localised and the population under investigation experiences greater vulnerability. In this article, we locate the source of this problem in a spatial scale mismatch between the essentialist framings of identity behind these generalised determinants of vulnerability and the intersectional, situational character of identity in the places where DRR interventions are designed and implemented. Using the Livelihoods as Intimate Government (LIG) approach to identify and understand different vulnerabilities to flooding in a community in southern Zambia, we empirically demonstrate how essentialist framings of identity produce this mismatch. Further, we illustrate a means of operationalising intersectional, situational framings of identity to achieve greater and more productive understandings of hazard vulnerability than available through the application of general determinants of vulnerability to specific places and cases.

## Introduction

It is now well-established that, to reduce vulnerability to shocks and stressors, we must understand risk not merely as exposure to hazards but as the outcome of such exposure as it is filtered through various social factors that shape individual sensitivity and the capacity to adapt to the impact of hazards (Cannon 1994; Cutter 1996; Cutter, Boruff & Shirley 2003; Cutter *et al.* 2008; Cutter & Finch 2008; Lee 2014; Turner, Kasperson *et al*. 2003; Turner, Matson *et al*. 2003; Wisner *et al.* 2004). Therefore, successful programmes for disaster-risk reduction (DRR) must look beyond generalised populations of concern to the intra-population characteristics that produce different vulnerability to particular hazards (Cannon 1994).

Whilst the literature on social vulnerability has furthered our understanding of vulnerability as complex and situational (see also Babugura, Mtshali & Mtshali 2010; Sultana 2010), in practice, DRR addresses the temporal and geographic specificity of vulnerability by operationalising generalised understandings of the social determinants of vulnerability. As Lee (2014:33) notes, this literature seeks general factors (especially preconceived social categories) that are seen to have an effect on vulnerability after many, if not all, disasters. Specific assessments and studies draw from these efforts, focusing their investigation on those general factors that are relevant to the case at hand (e.g. Bollin & Hidajat 2006; Cannon 1994; Comfort *et al.* 1999; Cutter *et al.* 2003; Cutter & Finch 2008; De Oliveira Mendes 2009; Dwyer *et al.* 2004; Ge *et al.* 2013; Lee 2014; Mustafa *et al.* 2011; Siagian *et al.* 2013; Tunstall, Tapsell & Fernandez-Bilbao 2007).

At the level of the regional or national assessment of vulnerability, such approaches often provide the resolution of information necessary to support policy decisions and broad planning needs (Krishnamurthy, Lewis & Choularton 2014; McLaughlin & Cooper 2010; Peduzzi *et al.* 2009). However, when such framings of vulnerability are applied to a project implemented in a particular place, they exhibit what Birkmann and Von Teichman (2010) call ‘spatial scale challenges’ where sources of information or conceptualisation do not align with the needs of those using the information. ^1^ Those concerned with vulnerability assessment for DRR have found that vulnerability indices based on general determinants of vulnerability produce incomplete explanations of the variance in vulnerability outcomes in particular places (e.g. Cutter 2006; Cutter & Finch 2008), declining explanatory power at increasing levels of social disaggregation (Schmidtlein *et al.* 2008) and decreasing precision in contexts of increasing vulnerability (Tate 2013). In short, broad indices of vulnerability suffer from attribution problems that become more acute as the level of analysis is localised and the population under investigation experiences greater vulnerability. This suggests that the framing of vulnerability inherent to these indices are inappropriate for the assessment of community-specific (project or activity level) vulnerability and carry real risks of misdiagnosing vulnerability in DRR programs and projects. This can result in interventions that miss the needs of some or all of the target population. In the worst cases, such misdiagnoses may serve to render these populations more vulnerable than before the intervention. Therefore, it is critical to the project of DRR that we identify and address the source of this spatial scale challenge.

In this article, we argue that the source of this challenge lies in DRR’s framing of identity. Specifically, we argue that DRR relies on an essentialist framing of identity that might serve DRR needs at high levels of spatial scale but fails to capture the specific vulnerability (and its causes) most relevant at the level of project implementation. Illustrating our argument though a case of vulnerability assessment for early warnings against floods in southern Zambia, we employ the Livelihoods as Intimate Government approach (Carr 2013, 2014). Through this approach, we intend to show, much as the contemporary literature on identity argues, that the identities relevant to vulnerability and DRR emerge at the *intersection* of the roles and responsibilities associated with different identities which are mobilised *situationally*, in the context of a particular hazard.

## Disaster-risk reduction, identity and the determinants of vulnerability

Nearly 10 years ago, Adger (2006:275) articulated the need to better establish causal links between characteristics which serve as general determinants of vulnerability and vulnerability outcomes. The mismatch between broad determinants of vulnerability and the information needs of project-level decisions in the DRR arena is a contemporary illustration of this problem. In DRR, this challenge stems from the essentialist framing of identity that serves as the foundation for assumptions about the relationship between generalised identities and individual vulnerability to particular hazards. For example, a portion of the hazards and DRR literature focuses on gender as a determinant of vulnerability, broadly linking women (and far less frequently, men) to greater vulnerability to particular hazards (Bradshaw 2004; Cutter *et al.* 2003; Davies *et al.* 2008; Dwyer *et al.* 2004; Khan 2012). Whilst this connection is often drawn from empirical evidence in specific cases, the literature on the determinants of vulnerability generalise these cases into sweeping statements about the links between gender and vulnerability. Such broad claims are only valid if gender is an essentialised category, one where its meanings and associated roles and responsibilities are very similar across contexts.

This broad framing of the relationship between identity and vulnerability runs contrary to a growing social-scientific literature in development and climate change adaptation which sees identity as situational and intersectional (see Carr & Thompson 2014 for discussion). Under this more contemporary framing of gender, associating the category ‘woman’ with vulnerability to a particular stressor at a particular place and time requires understanding women’s roles and responsibilities with regard to a particular activity upon which the stressor has an impact and then understanding how other aspects of identity (such as age) might further shape those roles and responsibilities.

Following contemporary feminist thought on identity, various authors in development and climate change adaptation have come to question the ways in which we identify the vulnerability and capacities of those who are vulnerable to hazards (Arora-Jonsson 2011; Carr 2008b; Demetriades & Esplen 2008; Harris 2006; Sultana 2010; Tschakert 2013; Tschakert & Machado 2012; Warner & Kydd 1997). For example, several authors (e.g. Arora-Jonsson 2011; Nelson, Meadows & Cannon 2002; Sultana 2010) question the empirical support for commonly stated claims that women, as a group, experience greater impacts from hydrometeorological hazards. Instead, they argue, disasters exacerbate existing patterns of discrimination that emerge through place-specific intersections of different identity categories, including age, socio-economic status, caste, ethnicity and religion. When framed in this manner, gender becomes a relational category that gains meaning through context as in the case of Hurricane Mitch where men’s mortality was higher than women’s, a fact that has been attributed to local constructions of masculinity in the context of emergencies (Demetriades & Esplen 2010:135, citing Röhr 2006). This framing of identity has become a common conversation in the wider development literature (Beetham & Demetriades 2007; Carr 2008b; Momsen 2009) and is emerging in the adaptation literature (Carr & Thompson 2014).

Despite clear theoretical and conceptual challenges to the prevalent framing of the nexus of identity and vulnerability in the DRR and hazards literatures, the bulk of on-the-ground assessments of local vulnerability to hazards draw heavily on general determinants of vulnerability and their essentialised framing of identity. Efforts to implement the livelihoods-vulnerability index (LVI) (Hahn, Riederer & Foster 2009), designed to integrate the exposure, sensitivity and adaptive capacity into vulnerability assessment at the community level, serve as examples of this challenge. The implementation of the LVI assesses vulnerability by measuring various determinants of social, health and resource access drawn from the literature but rarely validated at the project level. Further, the implementation of the LVI tends to focus at the household level as the smallest unit of analysis, obscuring intra-household vulnerability (e.g. Can, Tu & Hoanh 2013; Etwire *et al.* 2013; Hahn *et al.* 2009; Madhuri, Tewari & Bhowmick 2014). Even those that attempt to move beyond the level of the household impose assumed relationships between social categories/situations and increased vulnerability on the data collected during assessment. For example, in a comparative study of the vulnerability of two communities in Trinidad and Tobago, Shah *et al.* (2013:126) note that ‘… resilience and vulnerability are gendered by important norms in society’. In what appears to be a progressive framing of identity for the DRR literature, they further argue that the impact experienced by men and women and the ways in which they can respond are ‘… directly related to gender roles, relative socio-economic status and political power differentials’ (Shah *et al.* 2013:126). However, Shah and his co-authors do not embrace the promise of this early framing, treating gender as an intrinsic part of individual identity that is inextricably associated with different levels of vulnerability to climate variability and change.

In the following case of a flood-affected community in the Zambezi basin in Zambia, we show how the intersectional, situational framing of identity can resolve the particular spatial scale challenge that plagues the assessment of social vulnerability for DRR. We begin by comparing community-level patterns of livelihoods, vulnerability and the desire for different types of flood early-warning systems with those found when we disaggregate the population by the general determinants of vulnerability commonly employed in DRR. This exercise demonstrates that, in Kasaya, as in many other communities, community-level assessments of vulnerability are too coarse to identify different intra-community, flood-related vulnerabilities. Therefore, these assessments cannot explain intra-community differences in desired forms of early warning: A tailored approach is needed to inform action for flood-risk management. We then demonstrate that, whilst better than aggregated community analysis, the application of generalised determinants of vulnerability to the situation in Kasaya produces the same sorts of unexplained variance in vulnerability seen in other parts of the literature. Finally, we employ Carr’s (2013, 2014) Livelihoods as Intimate Government (LIG) approach to develop a situational, intersectional framing of identity in Kasaya that brings forth locally specific vulnerabilities. This exercise demonstrates the degree to which the use of essentialist framings of identity within generalised determinants of vulnerability can obscure both the actual vulnerabilities at play in a given context and the sources of those vulnerabilities, thereby costing project donors and implementers opportunities to address and ameliorate vulnerability.

## Methods

To illustrate a particular instance of the misalignment between concepts and information that challenges vulnerability assessment for DRR at the project level, this article draws on data collected as part of a USAID-funded project with the Red Cross Red Crescent Climate Centre and the Zambia Red Cross. This project explored the use of forecast-based data in DRR and the mobilisation of anticipatory response. ^2^ The discussion below is the product of data collected through 7 weeks of fieldwork in Kasaya, beginning on 13 February 2014 and ending on 4 April 2014. Before the main fieldwork was conducted, the authors, staff from the Red Cross Red Crescent Climate Centre and staff from the Zambia Red Cross conducted 2 weeks of pilot fieldwork to field test and tailor the LIG approach to Kasaya. Tozier de la Poterie then employed the approach on the ground with periodic remote consultation with the other authors.

**FIGURE 1:**
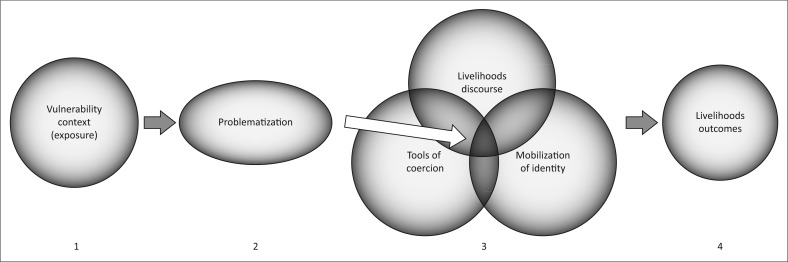
Conceptual map of the Livelihoods as Intimate Government approach.

LIG (Figure 1) frames livelihoods decisions as efforts by individuals and groups to negotiate the complex, shifting social, economic, political and environmental worlds they occupy (Carr 2008a, 2013, 2014). Therefore, livelihoods are much more than efforts to make a living. Livelihoods are better understood as efforts to govern the different factors that shape people’s everyday lives, setting oneself and others on a path toward one or more goals. Under LIG, these efforts are framed as taking shape at the intersection of three major factors, namely discourses of livelihoods, the mobilisation of identity and tools of coercion. Discourses of livelihoods are ‘… the language and actions that reflect different actors’ perceptions of the vulnerability context and the appropriate means of managing it in their everyday lives as they seek to achieve particular goals (income, empowerment, happiness, etc.)’ (Carr 2014:112). Mobilisation of identity refers to ‘… the roles and responsibilities associated with different subject positions within communities or households, such as those associated with men and women’ (Carr 2014:112). These identities serve to associate interests, roles and responsibilities with different individuals and groups. Tools of coercion are ‘… the locally legitimate institutional and social means by which some in a community or household can alter or affect the behaviours and choices of others’ (Carr 2014:112).

As in most livelihoods approaches, the LIG approach begins with an effort to establish the vulnerability context: the shocks, stressors and seasonality that people negotiate in their day-to-day lives. Initial work started with a desk study, which served as a foundation for interviews that then tested and deepened the team’s understanding of the challenges and opportunities experienced by different residents of Kasaya. Because these interviews sought to identify context-specific and identity-specific assemblages of stressors, we could not sample community members on the basis of identities *assumed* to be related to marginality and vulnerability in Kasaya. Instead, interviews were semi-structured, and the questions in the interviews evolved as patterns of vulnerability, livelihoods, and livelihoods decisions emerged. This approach to data collection required snowball sampling where Tozier de la Poterie asked informants, local translators and community leaders to identify others with specific vulnerabilities or challenges. Interviews continued until they no longer produced new lines of questioning or new responses to existing questions.

Fieldwork was conducted with the assistance of two translators, one male and one female, both of whom are residents of Kasaya and speak both Tonga and Lozi (languages common to the community). When possible, the interview team travelled to meet interviewees, and interviews were conducted at people’s homes, businesses, fields or gardens. However, those living farther away or in areas not easily accessible met the research team at the school or along the road.

This initial set of interviews served to identify *who* was vulnerable to *what* in Kasaya, shaping sampling for the rest of the research. LIG recognises that there is never a single vulnerability context at play in any population (Carr 2014). Instead, within a given population, there can be (1) distinct vulnerabilities where different members of a population are exposed to different events and trends and (2) differentiated vulnerabilities where the entire population shares exposure to events and trends, but different groups in that population have different sensitivities and adaptive capacities, producing different types of vulnerability to those events and trends (Carr & Thompson 2014). By looking for patterns of vulnerability identified in the course of this fieldwork, the team was able to divide the residents of Kasaya into four groups (Table 1), each comprised of individuals that reported similar assemblages of vulnerability.

**TABLE 1:** The four groups in Kasaya, as defined by their respective assemblages of vulnerability.

Group	Defining stressors
Group S (Severely constrained)	Lack of access to capital assets and lack of access to adequate water
Group C (Capital constrained)	Lack of access to capital assets
Group W (Water constrained)	Lack of access to adequate water
Group L (Least constrained)	No challenges related to either capital assets or access to water

After establishing the vulnerability context and the groups associated with particular assemblages of vulnerability, Tozier de la Poterie then conducted a new set of interviews, following up with the initial respondents and expanding the sample to a total of 109 individuals. In this round of interviews, she carefully interrogated discourses of livelihoods and constructions of identity, and she was able to identify evidence of tools of coercion. These data are discussed below. After the close of fieldwork, interview and observation notes were entered into MAXQDA, a qualitative analysis-support software, and coded for analysis. This coding allowed for the rapid retrieval of data, for example, on the number of individuals (or number of individuals with particular social and vulnerability characteristics) reporting a particular vulnerability or livelihoods activity. Further, it allowed the team to quickly identify relevant portions of interviews that provided a context for such information. After a preliminary analysis, the team removed five of the 109 interviewed individuals from the analysis because they were teachers temporarily stationed in the village and therefore with outlying and transitory experiences of the area. The discussions below result from the analysis of the remaining 104 individuals in the dataset, as well as corroborating observations from fieldwork.

It is important to note that a methodological challenge associated with the larger project from which this article is drawn created distinct challenges for the interpretation of data presented here. Because the larger project was trying to assess the relative importance of flooding in the lives of those in this community, continually focusing on flooding in every interview was likely to lead community members to identify flooding as the project’s focus. This might have caused interviewees to focus their discussions on this issue regardless of its actual importance in their lives. Therefore, the team decided to avoid this possible distortion in the overall dataset by limiting the prompting of interviewees regarding the utility of early warning. This had several effects. Firstly, it greatly limited the sample of those who discussed the utility of early warning as many residents did not mention early warning on their own. Secondly, as a result of our efforts to limit the introduction of bias towards flooding as a hazard into our sample, residents were not asked about issues of uncertainty in forecasts, except in a few cases, as specifying uncertainty bounds on forecasts might have affected residents’ views of the utility of early warning. Thirdly, not all residents have received early-warning information in the context of a flood and therefore might not yet have personal experience of the utility (or lack of utility) of a particular warning. Finally, whilst the team was trying to avoid biasing the data collected on vulnerability and flooding, in the analysis presented below, flooding is treated differently than other stressors because every resident was specifically asked about flooding before the end of their interview, potentially skewing the response rate with regard to this stressor. This did not bias the sample because it was asked only if the interviewee mentioned it first, or at the very end of the interview. If Tozier de la Poterie had to ask the interviewee about flooding, the question was posed in the context of other common stressors in the community, and residents generally saw the seasonal floods as an inherent part of the context. Questions about flooding specifically as a hazard in this area were therefore not seen as unusual by residents, and indeed nearly half of the residents interviewed raised flooding as a stressor without being prompted. By looking at percentages of respondents who mentioned flooding without being questioned directly about that hazard, we can gain a better sense of the number of residents who see flooding as a critical stressor and, therefore, the relative importance of this stressor vis a vis other stressors in the community.

## Community-level vulnerability in Kasaya

**FIGURE 2:**
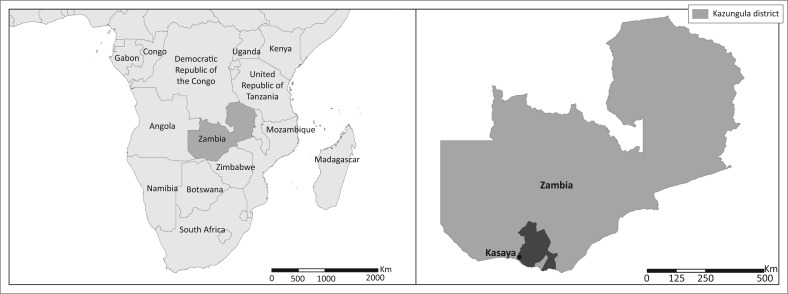
Locator map of Kasaya, in the Kazungula District of Zambia’s Southern Province.

The project from which this article is drawn was based in Kasaya, a community in Zambia’s Kazungula District and within the Zambezi basin, for a key reason – its exposure to seasonal flooding and occasional extreme floods (Figure 2). Kazungula, the district in which Kasaya is found, averages less than 800 mm of annual rainfall, and annual totals are variable (Swennenhuis 2012). At the same time, Kasaya is known for seasonal flooding as well as occasional severe floods, both of which are most common in January and February though they can start as early as October and last into March (Republic of Zambia 2007; Speranza 2010; Venkateswaran 2013). Whilst seasonal floods are annual events, major floods occur less frequently (the most recent occurred in 2006) and have an impact all members of the community. The 2005–2006 floods in the southern Province resulted in drowning; climate-sensitive disease outbreaks that affected humans, animals and plants; the destruction of agricultural crops; population displacements and damage to roads, houses and infrastructure (Republic of Zambia 2007). ^3^

**FIGURE 3:**
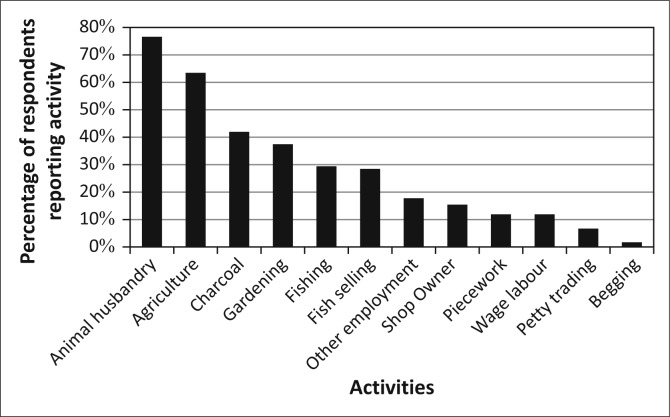
Livelihoods activities in Kasaya.

Kasaya has limited electrification, but due to its proximity to Livingstone, it has good primary-road and mobile-phone infrastructure (Ndiyoi & Phiri 2010). The population of Kasaya is comprised primarily of smallholders practicing subsistence agriculture. These farmers are reliant on maize cultivation for both income and for food security (Ndiyoi & Phiri 2010; Swennenhuis 2012). Relatively few residents of Kasaya have access to farming equipment (e.g. oxen or tractors). Farm labour is available to a limited extent as is the use of fertilizer or recycled seed. These factors, when coupled with the generally low fertility of the land, constrain agricultural production and result in low average yields of less than one ton per hectare for maize and significantly less for other crops (Swennenhuis 2012). Residents of Kasaya, and of the district as a whole, diversify their asset base by raising animals, catching fish and harvesting resources from forests and wetlands (Ndiyoi & Phiri 2010) (Figure 3).

It is difficult to establish the size of the population of Kasaya. The community is widely dispersed and recognised by residents to be divided into three parts: Mapani east, Simalaha south and Kasaya central (Venkateswaran 2013). Neither Kasaya as a whole nor any of its constituent parts are recorded in the 2010 Zambian Census. A Global Environment Facility report (Global Environment Facility 2013) lists 1022 residents in the Kasaya ‘catchment’. Tozier de la Poterie, while conducting fieldwork in Kasaya, held a community meeting where she was told that there were 4030 residents and 1300 households in Kasaya. The data at hand do not allow us to adjudicate between these claims. We note, however, that fieldwork was conducted in Kasaya central and, therefore, is focused most on the subset of the community living in this area.

Gaillard (2010; see also Blakie 1985) argues as follows:

Assets and resources essential in the sustainability or un-sustainability of livelihoods are conversely crucial in defining vulnerability. Such an intimate relationship between livelihood and vulnerability justifies that many people have no other choice but to face natural hazards to sustain their daily needs. (p. 221)

**FIGURE 4:**
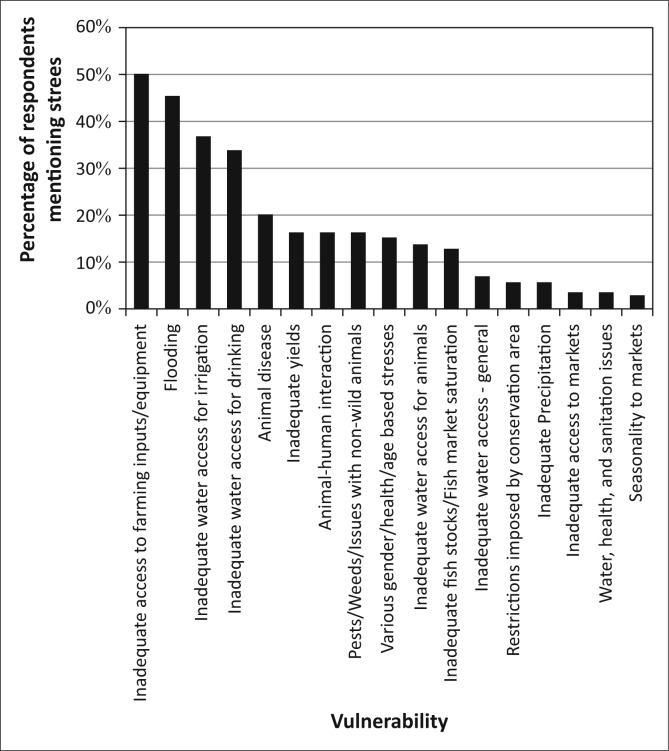
The overall vulnerability context of Kasaya, as reported by residents of the community.

As Figure 4 illustrates, the principal challenges identified by the residents of Kasaya are those where these natural hazards intersect with livelihoods activities: access to capital (equipment, capital and animals, principally for the purpose of agricultural production) and access to water (including drinking water, water for irrigation and water for animals). Flooding was mentioned without prompting by 45.2% of residents, suggesting that it is the second most commonly experienced stressor in the community. Animal disease and concerns for adequate yields also appear as concerns (both of which are impacted by flooding) though for only 20% or less of the population.

**FIGURE 5:**
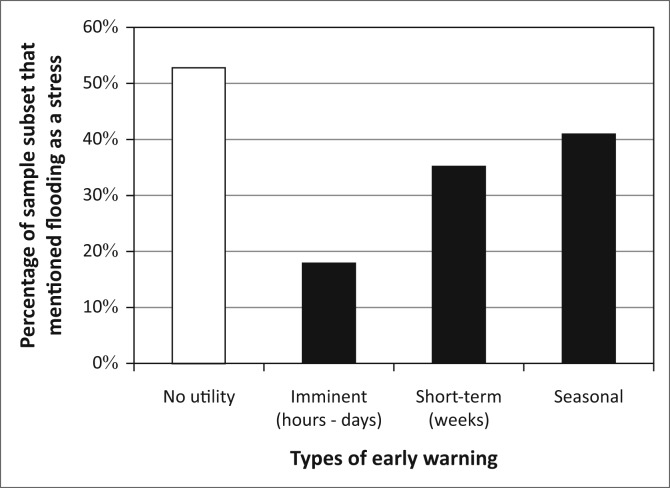
Types of desired early warning in Kasaya. Percentages sum to more than 100% because several residents expressed interest in more than one type of early warning.

Given the importance of flooding as a stressor to many in this community, it appears that the provision of accurate, timely flood early warnings could alleviate a significant vulnerability in the community. However, when we discussed the desire for early warning with residents who raised flooding as a challenge without prompt (*n* = 34), we found a range of interests (Figure 5). Notably, more than half of the community had no interest in early warnings. Those with an interest in early warnings expressed the desire for alerts across a range of different timescales. This less-than-uniform view of the challenges associated with flooding in this community suggests that Kasaya presents us with yet another situation where a shared biophysical stressor is translated into differentiated human vulnerability through social, economic and political processes (Cannon 1994; Carr & Thompson 2014; Wisner *et al.* 2004).

## Conventional approaches to the assessment of social vulnerability to flooding in Kasaya

**FIGURE 6:**
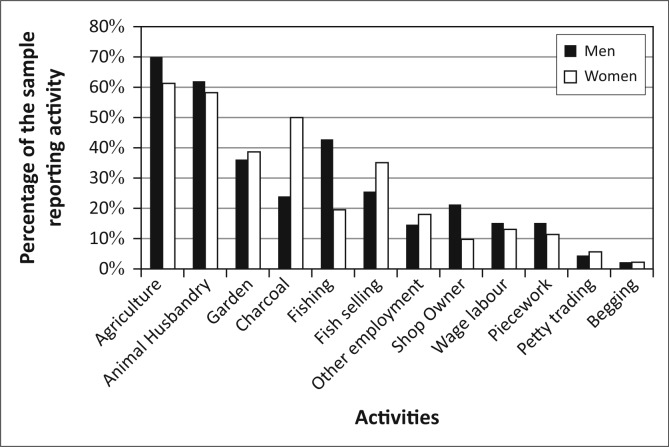
Livelihoods activities by gender in Kasaya.

As discussed above, amongst the categories most applied to the identification of social vulnerability in the DRR literature is gender (Bradshaw 2004; Cutter *et al.* 2003; Davies *et al.* 2008; Dwyer *et al.* 2004; Khan 2012). However, if we apply a basic gender lens to the population of Kasaya, we find relatively little with which to differentiate the livelihoods activities of men and women (Figure 6). Women produce charcoal at much higher rates than men, and men fish at much higher rates than women. However, residents did not mention either of these activities when talking about the impact of flooding. Therefore, the main impact that floods have on livelihoods appears to be through agricultural production and animal husbandry in which men and women participate at nearly the same level.

**FIGURE 7:**
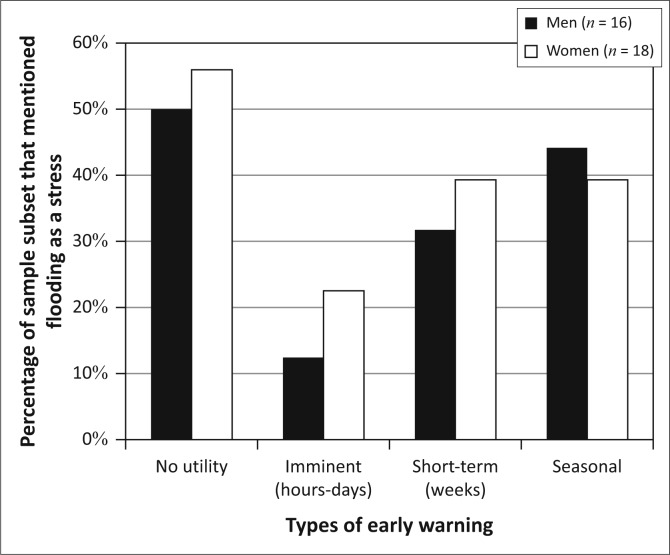
Types of desired early warning in Kasaya.

Men and women display very similar patterns of interest in early warning in Kasaya and largely mirror the community-level patterns of interest (Figure 7). This is not surprising, given that men and women have similar rates of participation in the different livelihoods activities observed in Kasaya.

**FIGURE 8:**
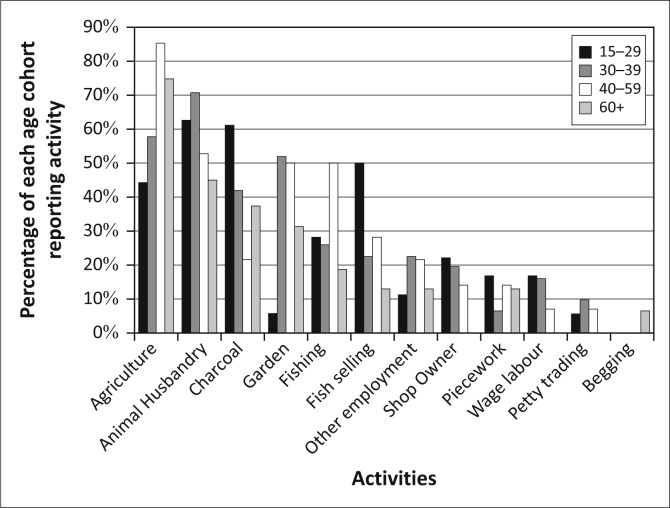
Livelihoods activities by age in Kasaya.

Another determinant of vulnerability commonly referenced in the literature is age where, for example, older people are assumed to become more vulnerable to various hazards over time (e.g. Cutter *et al.* 2003; Dwyer *et al.* 2004; Khan 2012). If we disaggregate the livelihoods activities in Kasaya by age cohorts, ^4^ we can see a few patterns (Figure 8). Firstly, as residents age, they become more engaged in agriculture and fishing. The rate of participation in animal husbandry rises until the age of 40 and then begin to decline. Producing charcoal and selling fish are most commonly performed by the youngest members of the community and then abandoned as they age.

**FIGURE 9:**
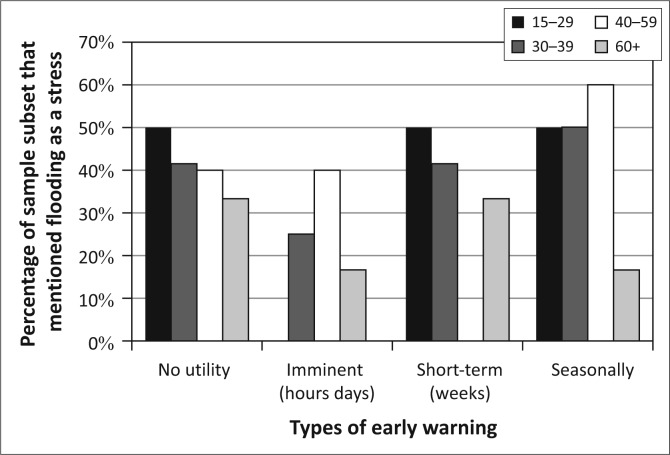
Types of desired early warning in Kasaya by age.

These differences in livelihoods partially explain the age-linked differences in the interest in early warning (Figure 9). The overall interest in early warning increases as residents age. This aligns with the increasing rate of participation in agriculture over the life course in Kasaya, an activity for which seasonal forecasts are of great utility and short-term forecasts are less useful as planted crops cannot be moved. However, this breakdown of the population does little to explain the declining interest in short-term (days to weeks) warnings as the population ages. It also does little to explain the pattern of changing interest in imminent (hours to days) warnings for floods. Whilst age certainly captures more about the character of social vulnerability in Kasaya than gender, it does not serve to explain all of the patterns of interest in early warning.

The foregoing application of general determinants of vulnerability^5^ to the particular situation of Kasaya empirically demonstrates the conceptual concerns raised at the outset of this paper. Some determinants (i.e. gender) typically associated with greater vulnerability do not produce any apparent differences in vulnerability outcomes as represented by interest in different early-warning timescales. Other determinants (i.e. age) only partially map onto different vulnerabilities. In summary, these general factors, when applied to a particular place experiencing significant vulnerability to a hazard (flooding), explain relatively little of the observed differences in the experience of that hazard. Such an analysis cannot inform a productive intervention that reliably addresses the different vulnerabilities that exist in this community.

## Identity, vulnerability, and the desire for early warning in Kasaya

Whilst the previous analysis shows the incomplete mapping of generalised determinants of vulnerability onto interest in flood early warning in Kasaya, it cannot show us what processes and patterns this incomplete picture obscures. In this section, we use the LIG approach to identify and interpret the social determinants of vulnerability to flooding in Kasaya, bringing into sharp relief the contributions of the essentialised framing of identity to this spatial scale mismatch.

### Establishing the vulnerability contexts of Kasaya

**FIGURE 10:**
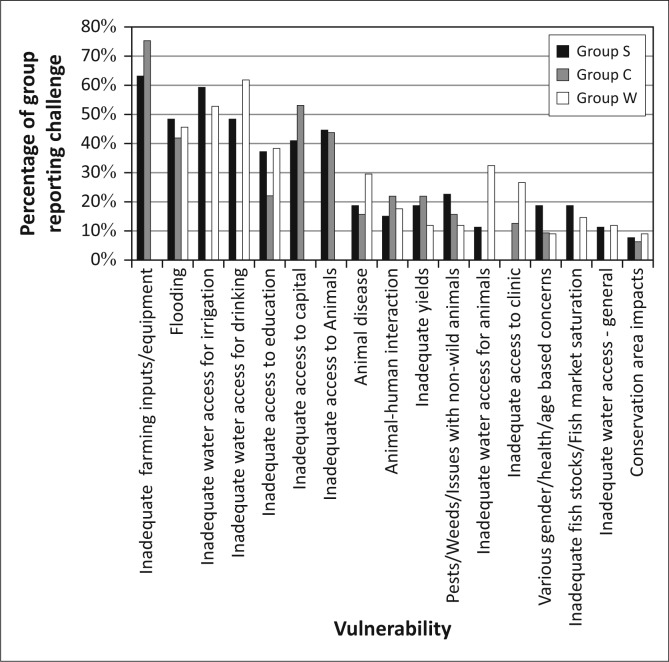
The different assemblages of vulnerability for the three groups in Kasaya.

The efforts to establish the vulnerability context of Kasaya found that the assemblages of vulnerability reported by residents can be clustered into four groups (Figure 10). Group S – *Severely Constrained* (*n* = 25, 23.8% of the sample) is comprised of residents who identified access to both capital and water as significant stressors in their lives. The five most commonly referenced challenges from respondents fell into these two broad categories. Roughly half (48.2%) of this group mentioned flooding as a challenge without prompting, suggesting that flooding is a less significant challenge than access to farming inputs and equipment or access to water for irrigation.

The assemblage of vulnerability for Group C – *Capital Constrained* (*n* = 31, 29.5% of the sample) is dominated by challenges of access to capital but did not reference access to water as a challenge. Forty-two percent of this group mentioned flooding as a challenge without being prompted. This suggests that, in this group, floods are less important stressors than most issues of access to capital.

Members of Group W – *Water Constrained* (*n* = 38, 36.2% of the sample) did not reference problems with access to capital, but they did reference challenges of access to water. In this group, the two most commonly referenced challenges, and three of the top four, were associated with access to water. Access to education was the third most commonly referenced challenge and was often linked to flooding and the distance that children had to travel through water. Forty five point seven percent of Group-W respondents mentioned flooding as a challenge without being prompted, suggesting that this group views flooding as a challenge on par with that of gaining adequate access to education for their children but less critical than issues of drinking water and access to irrigation.

A final group, Group L – *Low Constraints* (*n* = 11, 10.5% of the sample), did not mention either access to water or access to capital as a challenge. This last group was a combination of very elderly residents of the community who no longer have livelihoods responsibilities and a few individuals who were clearly wealthy relative to the rest of the community. Obviously, these are themselves very different groups with different challenges and opportunities, and the very small sample size for each precludes serious investigation into their challenges and opportunities. Therefore, in this article, we focus on the first three groups.

### The discourses of livelihoods in Kasaya

**FIGURE 11:**
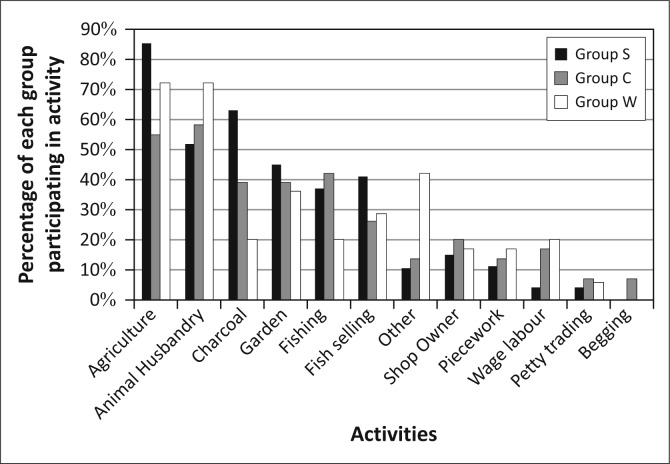
The different patterns of livelihoods activity for the three groups in Kasaya.

Given the close connection between livelihoods and vulnerability to hazards, it is not surprising that the livelihoods activities emphasised by these three groups are different (Figure 11). Group S is most heavily engaged in agriculture, charcoal production and animal husbandry, with gardening and various fishing-related activities also reported by a number of group members. Group C focuses heavily on a similar set of activities but with a far lower rate of participation in agriculture and charcoal production. Members of Group W put equal focus on agriculture and animal husbandry in their livelihoods and have the highest rate of participation in animal husbandry of the three groups. Group W also has the highest number of individuals working in non-farm jobs, as reflected in the miscellaneous piecework, petty trading and odd jobs listed by members.

**FIGURE 12:**
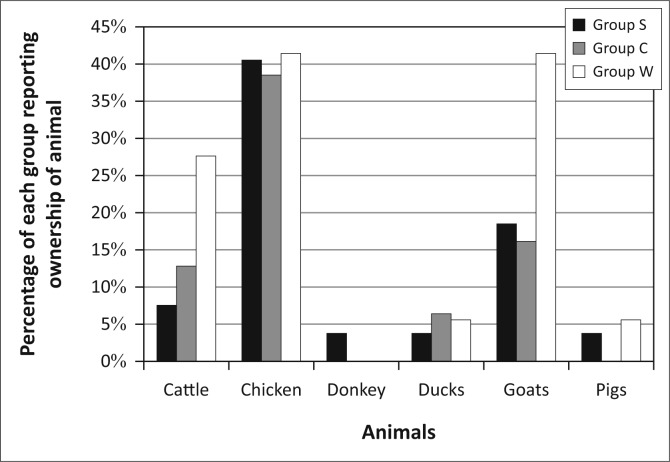
The different patterns of animal ownership for the three groups in Kasaya.

When we disaggregate animal husbandry by animals owned in each group (Figure 12), another set of differences emerges. Whilst chickens are raised by a similar percentage of each group, Group W dominates the ownership of cattle and goats. This is a critical difference between the groups as these animals are of higher value than chickens, and cattle also facilitate agricultural work like ploughing.

**FIGURE 13:**
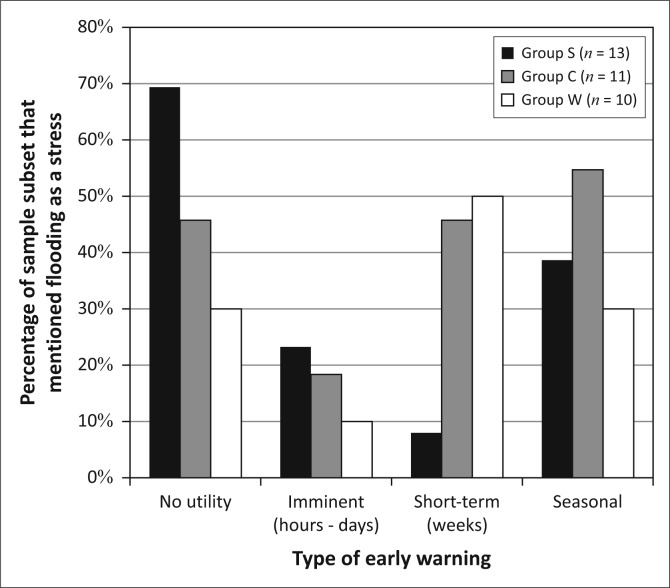
Answers of those who were asked what kinds of early warning would be useful, displayed by group.

Despite living in a single ‘community’, the residents of Kasaya neither share the same set of livelihoods activities nor experience the same assemblage of vulnerability. Because the three groups engage in different combinations of livelihoods activities and experience different assemblages of vulnerability, they have different interests in early warning (Figure 13). A closer examination of the decisions associated with these activities and the bases on which such decisions are made help to explain these divergent interests in early warning and how to address them.

Broadly speaking, rates of participation in various livelihoods activities in Kasaya reflect a continuum of desirability. This desirability was expressed by residents in interviews where they were asked to indicate which livelihoods activities they regarded as the best. For example, one senior man in the community (Interview 31) said that he wanted to stop fishing because it is harder and more dangerous work than gardening. A senior woman (Interview 84) argued that farming and gardening were better livelihoods activities than fishing because they provided food security for the household as well as a surplus to sell to finance her children’s education and needs. Thirty-nine residents expressed preferences for particular activities and rationales for that preference. Responses with regard to preference were so consistent across the community that Tozier de la Poterie stopped asking questions about the preferences for activities because such data would have added little to our understanding of the discourses of livelihoods in Kasaya.

The preferences in these statements were mirrored by the rates of participation in particular activities across the groups. For example, those with more assets and opportunities, such as those in Group W, had greater choices in the activities they undertook. Thus, what they prioritised provides a second source of data on activity preferences and therefore the desirability of different activities.

In Kasaya, agriculture is the most desirable activity, followed by gardening, fishing and, finally, charcoal production. Agriculture occupies this position despite being a source of subsistence, not income, for the vast majority of residents. The prioritisation of a subsistence activity above all others suggests a degree of conservatism in thinking about livelihoods in Kasaya where meeting the reproductive needs of the individual and household are most important, and other opportunities are secondary. Gardening, also an extremely desirable activity, represents a bridge activity between these conservative tendencies and the greater financial opportunity associated with market engagement. For some, gardening is a subsistence activity, but for a majority of gardeners, it is a source of income.

Fishing and selling fish are principally activities aimed at income generation. These activities are less desirable than gardening and agriculture because they are highly seasonal as government bans on fishing prohibit this work for portions of the year. Further, fishing is viewed as a dangerous activity, and whilst women are not prohibited from participating in fishing, most residents do not see this activity as suited for women. Remaining livelihoods activities, such as charcoal and piecework, are generally treated as means of earning money to address short-term challenges in the household or to meet the food needs of households when agricultural production or other activities do not. ^6^ On their own, they are not seen as adequate means of making a living in Kasaya. Therefore, as individuals accumulate assets and a greater ability to select their activities, we would expect a broad shift away from charcoal and fishing towards greater participation in agriculture and gardening. Piecework and other non-farm activities are difficult to interpret in this continuum. Nearly all households could use the access to income that such opportunities provide, but low-asset households may not have access to these opportunities due to a lack of basic tools or transportation needed to facilitate such employment.

Animal husbandry cannot be analysed alongside other livelihoods activities as it plays different roles depending on the animal in question, including serving as a means of conducting agricultural activities, storing wealth, financing other activities and meeting short-term household needs. Within animal husbandry, there is a continuum of desirability with cattle at the top, followed by chickens and goats and then on to ducks, pigs and donkeys. Animal husbandry is therefore desirable, but its desirability is highly animal-specific. Cattle are desirable because they facilitate agricultural production, overall the most desirable livelihoods activity in Kasaya. At the same time, cattle also provide food and income in the form of sour milk and act as a reserve of wealth for emergency situations. The only drawbacks to cattle ownership appear to be their high cost of purchase and their susceptibility to disease. Chickens and goats cannot play the agricultural role of cattle, but they serve as important stores of wealth that can be accessed to meet short-term household needs and, less commonly, to finance other livelihoods activities such as agriculture. Pigs and ducks are useful for generating income but only if the owner has the ability to move them to Kazungula or Livingstone for sale. Donkeys are a potential alternative to cattle for agricultural labour but do not provide milk or meat. Therefore, we expect that, as individuals accumulate assets, they will invest in chickens and other fowl, then goats and, finally, cattle.

These broad discourses of livelihoods reflect the desirability of particular activities and assets in Kasaya. They do not, however, explain the actual observed patterns of activity in these groups, which are shaped by *who* undertakes these activities and *why* individuals choose the activities they do. To better understand this, we must turn to the roles and responsibilities of different residents of Kasaya.

### Identity, roles, and responsibilities in Kasaya

Entry into more desirable livelihoods activities, such as gardening, or the acquisition of desirable assets, such as cattle, requires resources that often take time to acquire. This explains the partial mapping of livelihoods activities and the interest in early warning to age at the outset of this article (Figures 7 and 8). That mapping was partial, however, because of the ways in which gender shapes roles and responsibilities in Kasaya. Whilst community-level analysis of livelihoods, vulnerability and an interest in early warning showed no gendered patterns, when we conducted a gender analysis *within* these groups, we found that gender gains particular meaning in the context of asset ownership and access, particular livelihoods activities and the particular stresses presented by floods.

In Kasaya, men and women play different roles in their households and in livelihoods activities. Table 2 lays out the roles and responsibilities reported by men and women when they were asked about the attributes of a ‘good man’, ‘good husband’, ‘good woman’ and ‘good wife’. The table speaks to the community’s general views on gender roles and responsibilities. When asked directly about economic decision-making in the household, men and women often responded that the husband and wife sit together and come to a decision. However, looking at the responsibilities of men and women, it is clear that such consultations are not egalitarian as men clearly make more decisions than women and have fewer responsibilities for the everyday maintenance of the household.

**TABLE 2:** Gender roles and responsibilities referenced by residents of Kasaya in interviews.

Gender	Agriculture	Children	Household management	Household responsibilities	Livelihoods activities
Women	Agricultural decisions with husband	School fees alone	Household economic decisions with husband	Purchase goods for the house	Selling milk
	Does not make agricultural decisions	School fees with husband	Economic decisions for her activities	Purchase food	Selling fish
	Weeding	Childcare	-	Buy clothes	Cut thatch
	Harvesting	School uniform	-	Housework	-
	Planting	Children’s expenses	-	Medical expenses	-
	Tending fields	-	-	Collect water	-
	Ploughing	-	-	-	-
Men	Planting decisions alone	School fees by himself	Economic decisions with wife	Hospital fees	Care for cattle
	Planting decisions with wife	School fees with his wife	Economic decisions for his activities	-	Fishing
	Ploughing	School uniforms	Economic decisions by himself	-	Building fences/housesHard labour

Amongst Kasaya’s married residents, men manage the household, from broad livelihoods to specific agricultural decision-making. In terms of direct responsibilities to the household, men are responsible for children’s’ schooling, hospital fees, the construction of houses and the construction of fences. These roles speak to men’s broader responsibility to earn cash and conduct ‘heavy labour’ that residents see as too difficult for women. Women are responsible for nearly everything else, from caring for children to purchasing needed household items. For example, in agriculture, men are responsible for ploughing (heavy labour) and most major decisions whilst women are responsible for all other (light) activities. In short, women appear to have a greater responsibility for household reproduction than men, who broadly enable these activities whilst incurring few specific responsibilities. ^7^

### Tools of coercion in Kasaya

Identifying and elaborating the tools of coercion in any context is challenging as any investigation necessarily engages local politics (and even intra-household politics) that can take extensive time and relationship-building to unearth. In Kasaya, our understanding of the tools of coercion is limited. Generally, however, the tools of coercion applied to men and women are different in character. Men are subject to greater pressure via their reputation. For example, a wealthy man with cattle would experience a loss of reputation if, when he was finished ploughing, he did not allow poorer residents to use those cattle. Further, men who failed to provide the materials and assets necessary to meet household needs would also suffer the impact on their reputation. Discussions with men did not uncover why a loss of reputation might be specifically undesirable. Women, in contrast, reported being subject to more material forms of discipline. For example, some women noted that if they did not consult their husband on spending decisions, they could be beaten. Further, if women were divorced, they often had to return to their family villages. The strength of such coercion was evident in the response of one 25-year-old woman, who, when asked about spending decisions, claimed that she had never thought of spending money without asking and had no idea what would happen if she did. Therefore, whilst we cannot concretely apply specific tools of coercion to the livelihoods decisions of residents described in this article, it is clear that such tools exist and therefore operate in the background as a means of regulating decisions and behaviour. Whilst a fuller understanding of these tools might provide greater explanatory resolution for the choices that different individuals make in their livelihoods, knowing that residents of this community make decisions with the potential for social sanction serves partially to explain the consistency in these decisions amongst those who share particular roles and responsibilities.

## Groups, livelihoods and vulnerability in Kasaya

When we link discourses of livelihoods to gendered roles and responsibilities, recognising the existence of sanctions for those who transgress these expectations, patterns of vulnerability and of interest in early warning emerge. These patterns take shape at the level of groups with different degrees of access to assets. These patterns, which are otherwise invisible at the community level, illustrate the spatial scale challenge created by the application of essentialist framings of identity, operationalised as general determinants of vulnerability, to the place-specific ways in which identity and livelihoods are linked to produce vulnerability.

### Group S: Severely constrained

**FIGURE 14:**
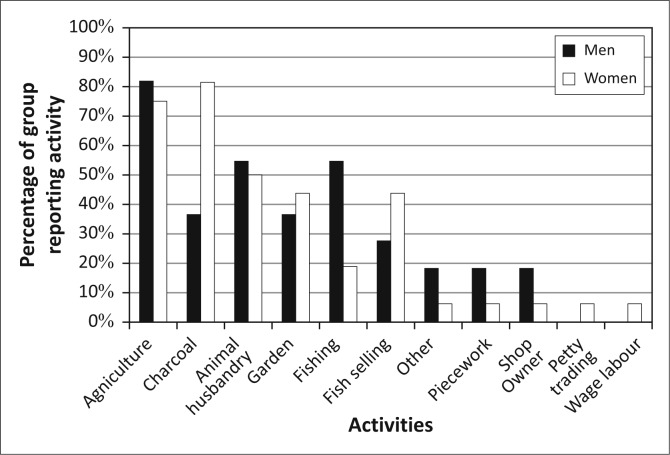
Livelihoods activities by gender in Group S.

Roughly the same percentage of men and women in Group S engage in agriculture (Figure 14). Whilst animal husbandry is sensitive to flooding, these households principally own fowl, which are easily transported out of harm’s way. Finally, men are far more engaged in fishing than women. Because fishing is not heavily impacted by flooding and is indeed banned during much of the flooding season, these men are less sensitive to floods and likely to have a greater adaptive capacity to address flooding than the agriculturalists in this group.

**FIGURE 15:**
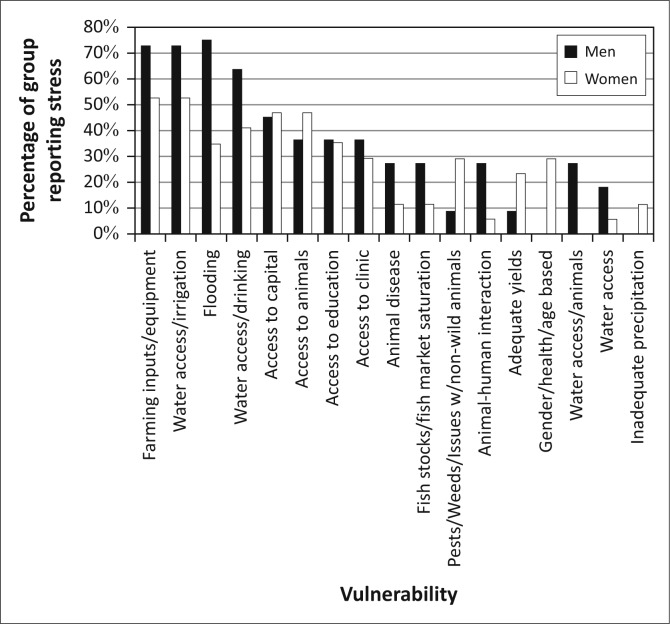
The gendered vulnerability context of Group S.

**FIGURE 16:**
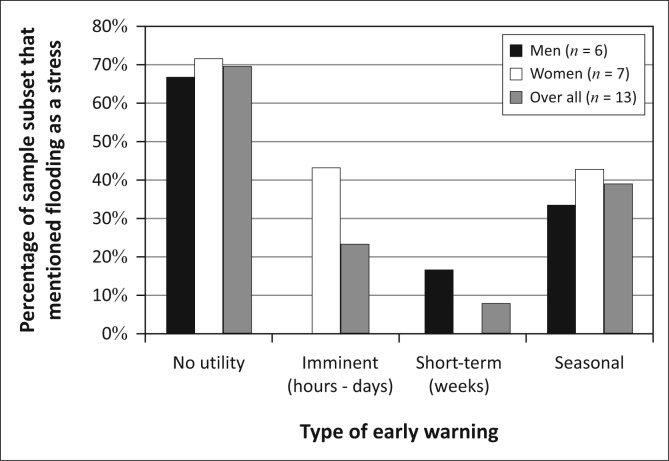
Answers of those in Group S who were asked what kinds of early warning would be useful, broken up by gender.

The gendered vulnerability context of Group S reflects these gendered patterns of livelihoods and the place of flooding in the shocks and stresses managed by men and women (Figure 15). In Group S, 75% of the men mentioned flooding as a stressor without being prompted, as opposed to only 35.3% of women. Therefore, for men in this group, flooding is the single most important stressor they face whereas for women flooding and access to education are tied as the sixth most important stressor. However, nearly 70% of all men *and* women in this group expressed no interest in early warning (Figure 16). This suggests that, for the majority of people in this group, flooding is less important than many challenges related to access to water and access to capital. Women’s low overall interest in early warning can at least partially be attributed to their high rates of participation in relatively flood-insensitive activities like charcoal production and fish selling. Men and women focus much of their limited interest in early warning on seasonal forecasts as their adaptive capacity with regard to agriculture is largely limited to the choice of fields to plough and work. Men’s interest in imminent warnings represents the only other decision over which they have control, that of when to move out of the way of a flood to avoid the loss of assets and housing.

### Group C: Capital constrained

**FIGURE 17:**
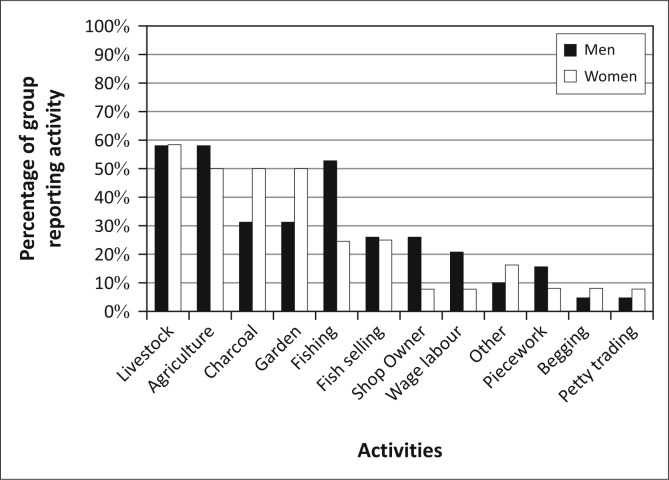
Livelihoods activities by gender in Group C.

In Group C, women participated in charcoal and gardening more frequently than men, but the rate of participation in charcoal production was much lower than in Group S (Figure 17). This may be because these women have greater access to water resources that enable gardening, allowing them to leave the undesirable labour associated with charcoal. This constitutes what most women in Kasaya would feel was a positive change in livelihood. However, because the overall suite of livelihoods activities for women in Group C de-emphasises flood-insensitive activities like charcoal production and fish selling relative to Group S, women in Group C are somewhat more exposed and thus sensitive to flooding. Further, because this activity is literally rooted in place, and these individuals are not the wealthiest members of the community, these women have limited adaptive capacity to address the impact of floods on their activities. Men in Group C participated in agriculture at the same rate as women, but their overall livelihoods incorporated much greater attention to fishing than in Group S. Participation in this activity makes them less sensitive to flooding than those whose livelihoods are limited to agriculture or gardening. Finally, their greater participation in non-farm activities probably diversifies their livelihoods, providing adaptive capacity that further insulates them from the impact of flooding.

**FIGURE 18:**
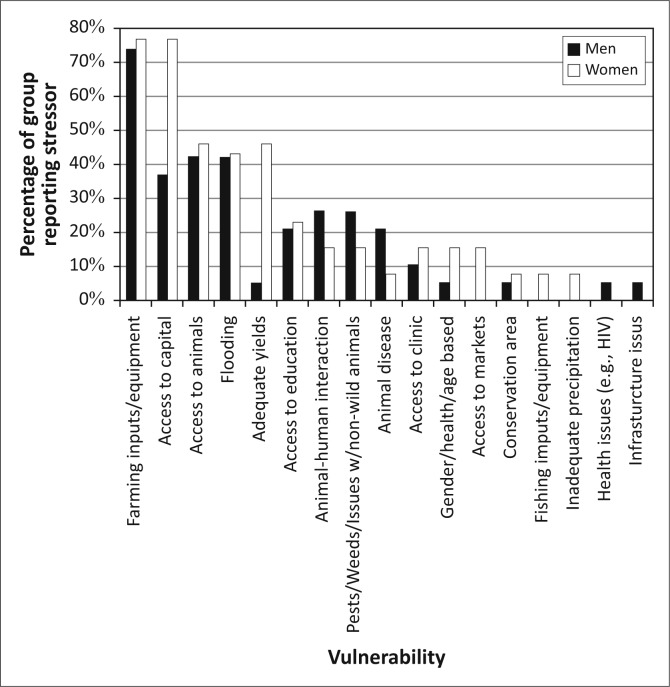
The gendered vulnerability context of Group C.

**FIGURE 19:**
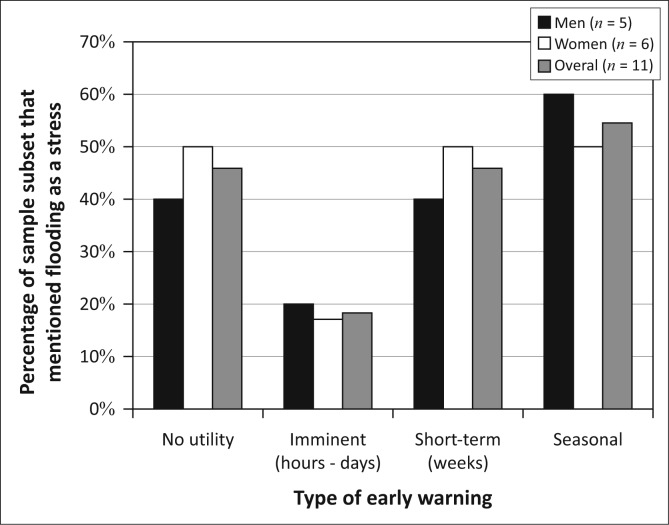
Answers of those in Group C who were asked what kinds of early warning would be useful, broken up by gender.

These gendered patterns produce a different gender-disaggregated vulnerability context than that seen in Group S (Figure 18). Firstly, in Group C, quite similar numbers of men and women identified flooding as a stressor without being prompted (42.1% of men and 41.7% of women). This suggests that flooding is the third most important stressor for men in this group and the fifth most important stressor for women. Secondly, men and women in Group C were nearly equally concerned with the need for farming inputs and equipment. They were also similarly concerned with access to animals. The gendered patterns of participation in these core livelihoods areas seen in Group S were largely absent in this group. In part, this is because the entire group has adequate access to water for its needs. Therefore, the principal limiting factor for agricultural production and gardening is access to inputs, equipment and animal traction. Early warning does not address any of these challenges. Men’s roles and decision points in agriculture have not changed from those in Group S. Further, they were heavily focused on fishing, animal husbandry and various small wage-paying jobs as means of meeting their obligations to the household. These men owned few animals, and those they owned were usually fowl or goats. Despite the fact that men in Group C had the lowest level of engagement with agriculture and gardening of any group in the community, at least in part because of the challenges presented by obtaining needed equipment and inputs without access to adequate capital, they had much higher levels of interest in seasonal warning than those in Group S (Figure 19). The rising interest in short-term warning may have to do with the need to harvest whatever can be salvaged in the face of a flood. Unlike those in Group S, many of these men have access to fishing boats that can be used to salvage crops during floods. Women’s rising concern for flooding is explained by their decreased participation in relatively flood-insensitive activities like charcoal production and fish selling, compared to Group S. Their increased interest in short-term warnings is difficult to interpret and may be related to a greater stock of household assets to be saved in the event of floods, compared to Group S.

### Group W: Water constrained

**FIGURE 20:**
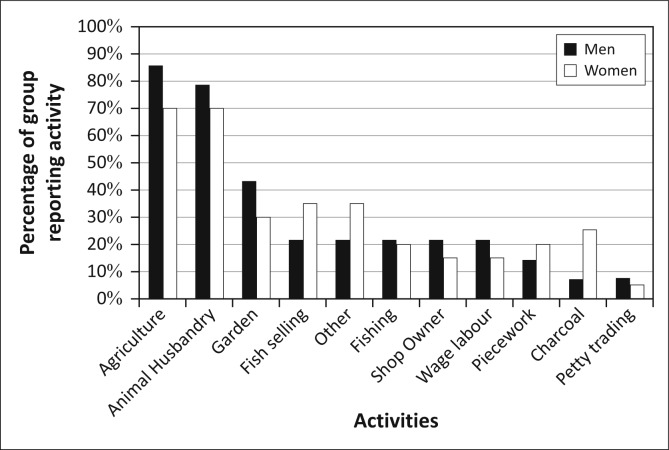
Livelihoods activities by gender in Group W.

The livelihoods activities of Group W, whilst founded on agriculture and animal husbandry, are in fact distinct from those in the other groups (Figure 20). Group W combined rates of participation in agriculture and animal husbandry similar to Group S with rates of participation in non-farm activities similar to those in Group C. Men in Group W owned nearly all of the cattle in Kasaya. These men provide the animal traction that all other residents in the community need to farm. Men in Group W lend these animals to family, improving and reinforcing their status in the family. They can also build their income or their standing in the community by renting or lending their cattle for ploughing and by lending their cattle to others on a semi-permanent basis. More so than in Groups S and C, the men in Group W have social status that extends beyond the household to the wider community, a responsibility that has developed in the context of their greater wealth and ownership of cattle.

Women in Group W reported the lowest rate of participation in charcoal production of any women in Kasaya. This, when combined with a relatively low rate of participation in fish selling (compared to Group S) and their high rates of participation in agriculture (compared to Group C), makes them the most exposed and sensitive to flooding in Kasaya. Their vulnerability to the impact of floods might be mitigated, to some extent, by the adaptive capacity that goes along with their participation in non-farm activities and by the fact that, as members of the wealthiest households, they are likely to be farming the most preferred (elevated) land in the area.

**FIGURE 21:**
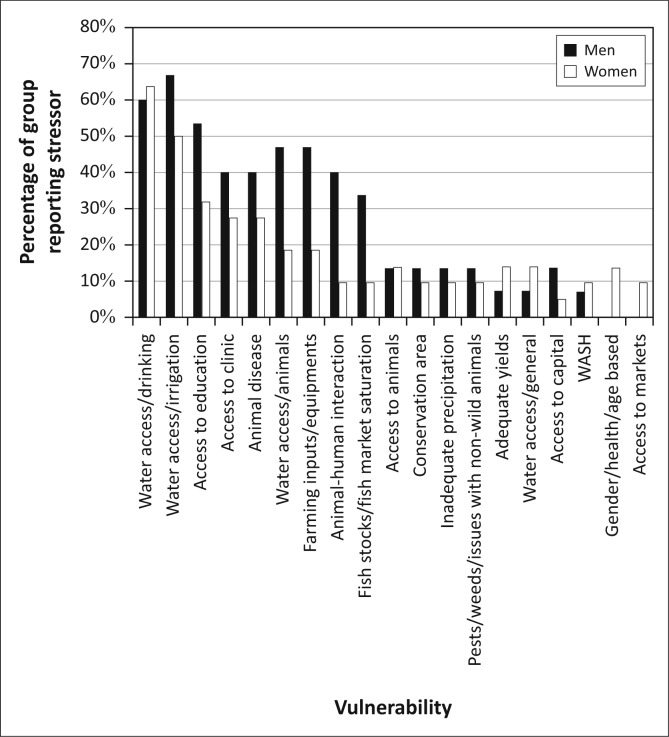
The gendered vulnerability context of Group W.

These forms of gendered livelihoods produced slightly gendered patterns of vulnerability in this group (Figure 21). The rates of unprompted reference to flooding in Group W were very similar to those in Group C, at 41.7% of men and 42.9% of women. This suggests that, in this group, flooding is the fifth most significant stressor for men and the third most significant for women. Issues of access to water dominated in both men’s and women’s vulnerability contexts, and concerns for access to water disaggregated by gender in somewhat predictable ways. Given their high rate of animal ownership, men were more concerned with adequate water for animals, especially cattle, whilst women were more concerned with water for household use. In this group, there appears to have been a pivot in the vulnerability context. Group W is the only group where men were more concerned about access to education than women. Whilst providing for the education of their children is an expected responsibility of all men in Kasaya (see Table 2 above), it may be that, in this group, they are sufficiently secure in their access to needed resources to begin to prioritise this responsibility.

These roles and vulnerability are translated into gendered interests in early warning (Figure 21). The vast majority of men in Group W saw early warning as useful. This interest was greatest in either seasonal warnings that inform planting decisions or short-term warnings in the order of weeks in advance of a flood. In both cases, levels of interest were similar to those seen in Group C. In Group W, men derive their status and livelihoods in large part from the ownership and use of cattle. These households generally have access to better (more elevated) farmland and may therefore be able to select fields to avoid seasonal floods. Whilst grazing land is communal, much of that land can be submerged in a major flood. Therefore, an individual owning cattle or goats (and these animals are nearly exclusively owned by men) will likely need to identify high ground onto which he can move these animals for safety and grazing. As much of the high ground in the area is already farmed, finding such a place likely takes negotiation and cannot be arranged in a few hours or days. Short-term warnings will come too late to salvage these animals.

Though the women in this group are exposed and sensitive to flooding, their adaptive capacity (and that of their households) allows them to be somewhat less interested in early warnings than women in Group C. As in Group C, these women had a great deal of interest in short-term warnings, likely because of their husbands’ need to move cattle out of the path of floods. Women’s lower interest in seasonal warning reflects their participation in NFE, which insulates their income against flood shocks. It may also be linked to both their households’ better access to elevated land, which limits their personal experiences of flooding, and the fact that women in these households generally do not decide what fields to plant.

## Discussion

The preceding analysis demonstrated how the spatial scale challenge at the heart of vulnerability assessment for DRR is anchored in the essentialist construction of identity that predominates in the hazards and vulnerability literature. Analysis using common generalised determinants of vulnerability left a great deal of unexplained variance between these determinants and reported assemblages of vulnerability. However, when we dug into the local construction of livelihoods decision-making, which provides the situational, intersectional context in which identity takes meaning with regard to vulnerability, we uncovered intra-community vulnerabilities to flooding that produced variable interest in early flood warnings.

**FIGURE 22:**
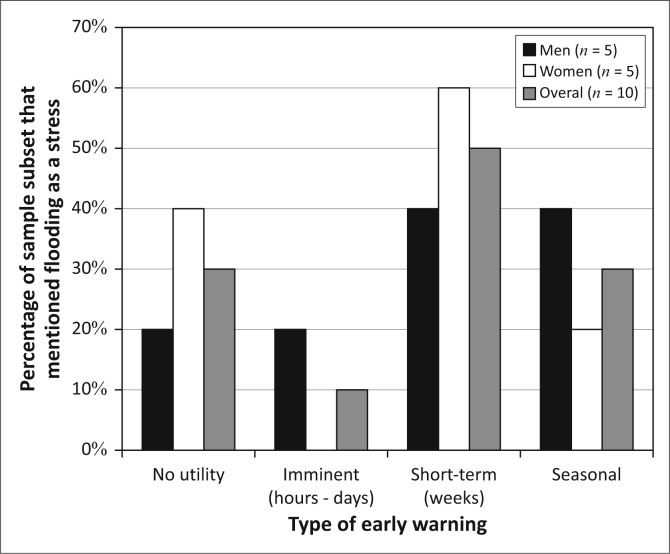
Answers of those in Group W who were asked what kinds of early warning would be useful, broken up by gender.

**FIGURE 23:**
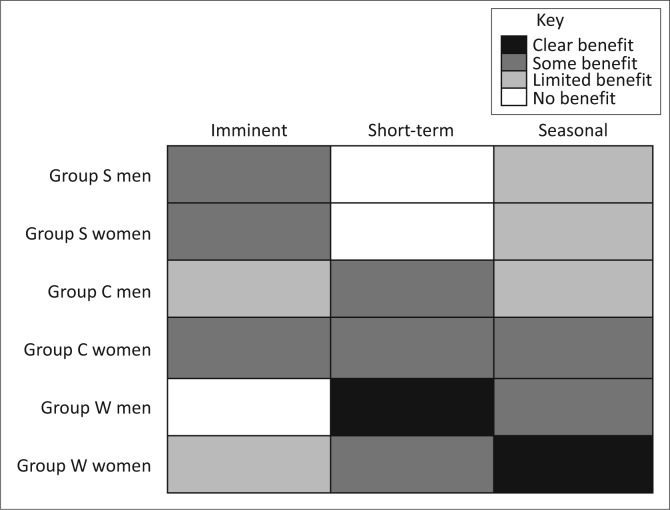
Graphical representation of who benefits from different timescales of early warning and the degree to which they benefit.

Figure 22 represents our understanding of who benefits from what time frame of early warning, based both on individual responses and our understanding of livelihoods decision-making enabled by the framing of vulnerability around situational, intersectional constructions of identity. What this analysis reveals is a potentially interesting pattern. As households gain assets and improve the security of their livelihoods, their interest in flood early warnings shifts from short to longer-term warnings. Imminent warnings appear to be of greatest interest for the poorest and most asset-challenged members of the community, perhaps because their limited access to assets greatly limits the sorts of decisions they can make to those that might save lives or material assets in the face of an impending flood. Short-term warnings benefit those who own cattle, the wealthiest in the community, the most. Seasonal warnings benefit a wider range of residents but can do little for the land-constrained who do not have other options for planting. The patterns of interest in Figure 23 present a potentially important characteristic of DRR worth pursuing, not only to improve the reach of DRR efforts within a given community but also to establish better connections between DRR and climate change adaptation initiatives. As individuals and households accumulate assets and status, their livelihoods security increases, and their DRR interests start to take on timescales that approach adaptation planning rather than reactive, short-term information needs. This is further evidence that the close connection between DRR, adaptation to climate change and development require greater attention for successful programs in all three arenas (Schipper 2007; Schipper & Pelling 2006).

## Conclusion

This article, like many before it, demonstrates that vulnerability to a hazard is produced through local social processes and practices that shape who conducts what activities and therefore their exposure, sensitivity and adaptive capacity with regard to that hazard. However, as others identified and as we demonstrated empirically, at the project level, the most common approach to assessing social vulnerability, namely stratifying a population by general determinants of vulnerability, is deeply flawed. We have demonstrated that the spatial scale challenge that emerges in the mismatch between general determinants of vulnerability and their limited explanatory power at the DRR project or activity level lies at least in part in DRR’s essentialist framing of identity. This framing elides the situational, intersectional character of identity and its attendant roles and responsibilities that produce shifting, differentiated forms of vulnerability to particular hazards at particular places and times. When we applied a situational, intersectional understanding of the identities, roles and responsibilities associated with different vulnerabilities, and therefore different DRR needs, the result was a more nuanced understanding of vulnerability in Kasaya than possible with any assessment based on essentialist, predetermined factors. Such factors, whilst perhaps valid for vulnerability analysis at high levels on the spatial scale of social vulnerability, risk missing the mark at lower levels of the scale such as that of communities or households. Reframing our approach to identity in the context of vulnerability assessment is critical in the present as it allows us to tailor available early warnings to the individuals and groups that can benefit from early actions at different timescales. It will become more valuable in a future where global environmental change is likely to increase the magnitude and frequency of extreme events.

## Footnotes

1 In this article, we draw on the long-held discussion of scales and levels of analysis in the literature and practice of DRR (for example Birkmann 2007; Birkman & Von Teichman 2010; Blackburn 2014; Fekete, Damm & Birkmann 2010) to define both scale and level. Following Fekete *et al.* (2010:731), we treat scale as ‘… the vertical axis along which any objects of interest are ranked, like on a ruler’ whilst ‘[*l*]evel is a fixed rank or horizontal layer on a scale. On this level, all units are of the same categories’.

2 For an overview of current knowledge about changing climate risk as well as the observed and projected impact for Zambia, see Gannon *et al.* (2014).

3 Whilst floods are a challenge for the residents of Kasaya, they also create opportunities. For example, many members of the community noted that seasonal flooding brings fish to the immediate environs of the village, enabling another livelihoods activity while the floodwaters persist. Further, many members of the community noted that the floodwaters are critical to agricultural livelihoods and animal husbandry as they provide much of the water for these activities in the otherwise arid environment of Kazungula.

4 Data are broken into these uneven cohorts due to the sample sizes in each. To gain meaningful numbers within each age cohort, we had to lump all respondents between 18 and 30 into a group as we had to do with all respondents between 40 and 59 and with all those above 60. This division loosely aligns with the stages of the life course for men and women in Kasaya, from unmarried to early married adults, established adulthood, middle age, and old age.

5 Our analysis considered several identity categories that might have shaped vulnerability to flooding, including language. There were not enough Tonga speakers in the community or our sample to identify meaningful differences in livelihoods or vulnerability associated with language. We did not analyse the role of religious affiliation in the production of vulnerability, for whilst the residents of Kasaya claimed affiliation with a range of Christian denominations, the residents did not link these affiliations to livelihoods or vulnerability.

6 Charcoal production also suffers from the challenge of being restricted by the state. Charcoal producers can have their products confiscated, leaving them without any income.

7 Whilst these general representations of men’s and women’s roles and responsibilities hold across the community, women heading households in Kasaya often take on ‘men’s’ duties. For example, in Table 2, it was women heading households who claimed that they were responsible for school uniforms and school fees, tasks that would otherwise be classified as men’s responsibilities.
